# Supplementary material for: Modified Protein-Water Interactions in CHARMM36m for Thermodynamics and Kinetics of Proteins in Dilute and Crowded Solutions
Source: Molecules. 2022 Sep 5;27(17):5726. doi: 10.3390/molecules27175726 (PMC9457699; doi:10.3390/molecules27175726)
Supplement: Supplementary file 1 [file molecules-27-05726-s001.zip › molecules-1834934-supplementary.pdf]

Supporting Information for

Modified Protein–Water Interactions in CHARMM36m for Thermodynamics and Kinetics of Proteins in Dilute and Crowded Solutions

Daiki Matsubara[1], Kento Kasahara[1,2], Hisham Dokainish[3], Hiraku Oshima[1], and Yuji Sugita[1,3,4,*]

[1] Laboratory for Biomolecular Function Simulation, RIKEN Center for Biosystems Dynamics Research, Kobe, Hyogo, Japan.

[2] Division of Chemical Engineering, Graduate School of Engineering Science, Osaka University, Toyonaka, Osaka, Japan.

[3] Theoretical Molecular Science Laboratory, RIKEN Cluster for Pioneering Research, Saitama, Japan.

[4] Computational Biophysics Research Team, RIKEN Center for Computational Science, Kobe, Hyogo, Japan.

[*] To whom correspondence may be addressed. Email: Yuji Sugita (sugita@riken.jp)

Scheme of computing diffusion coefficients of villin

It is known that the diffusion coefficient has a system size dependency, which arises from the artifacts of the periodic boundary condition (PBC). The expression of the corrected diffusion coefficient was proposed by Yeh and Hummer [1] as

$$D = D_{\mathrm{MSD}} + \frac{k_{\mathrm{B}}T}{6\pi\eta L}\left(\xi - \frac{4\pi R_h^2}{3L^2}\right), \tag{1}$$

with $\xi = 2.837$. Here, D_{MSD} is the diffusion coefficient evaluated from the slope of the mean square displacement (MSD) as

$$D_{\mathrm{MSD}} = \lim_{t\to\infty} \frac{1}{6t} \mathrm{MSD}(t). \tag{2}$$

k_B, T, L, η, and R_h are the Boltzmann constant, absolute temperature, box length of the system, solvent viscosity, and hydrodynamic radius of villin, respectively. Let us define the second term of Eq. (1) as D_{PBC}. The value of R_h is taken from the HYDROPRO [2] estimation as 13.86 Å reported in the previous study [3]. The viscosity of the TIP3P water, $\eta_{\mathrm{TIP3P}} = 0.35$ cP [4], is used for the dilute solution, while the viscosity of the crowder solution is estimated as

$$\eta_C = \eta_{\mathrm{TIP3P}}(1 + 2.5\phi), \tag{3}$$

where ϕ is the volume fraction of the crowders determined from R_h. The TIP3P water model is known to underestimate the water viscosity ($\eta_{\mathrm{expt}} = 0.89$ cP [5]), leading to the overestimation of the diffusion coefficient. According to the previous study [3], the viscosity correction described as

$$D' = D\frac{\eta_{\mathrm{TIP3P}}}{\eta_{\mathrm{expt}}}, \tag{4}$$

is employed.

References

1. Yeh, I.-C.; Hummer, G. System-Size Dependence of Diffusion Coefficients and Viscosities from Molecular Dynamics Simulations with Periodic Boundary Conditions. *J. Phys. Chem. B* **2004**, *108*, 15873–15879,

doi:10.1021/jp0477147.

2. Ortega, A.; Amorós, D.; García de la Torre, J. Prediction of Hydrodynamic and Other Solution Properties of Rigid Proteins from Atomic- and Residue-Level Models. *Biophys. J.* **2011**, *101*, 892–898, doi:10.1016/j.bpj.2011.06.046.
3. Nawrocki, G.; Wang, P.; Yu, I.; Sugita, Y.; Feig, M. Slow-Down in Diffusion in Crowded Protein Solutions Correlates with Transient Cluster Formation. *J. Phys. Chem. B* **2017**, *121*, 11072–11084, doi:10.1021/acs.jpcb.7b08785.
4. Feller, S.E.; Pastor, R.W.; Rojnuckarin, A.; Bogusz, S.; Brooks, B.R. Effect of Electrostatic Force Truncation on Interfacial and Transport Properties of Water. *J. Phys. Chem.* **1996**, *100*, 17011–17020, doi:10.1021/jp9614658.
5. Korson, L.; Drost-Hansen, W.; Millero, F.J. Viscosity of Water at Various Temperatures. *J. Phys. Chem.* **1969**, *73*, 34–39, doi:10.1021/j100721a006.

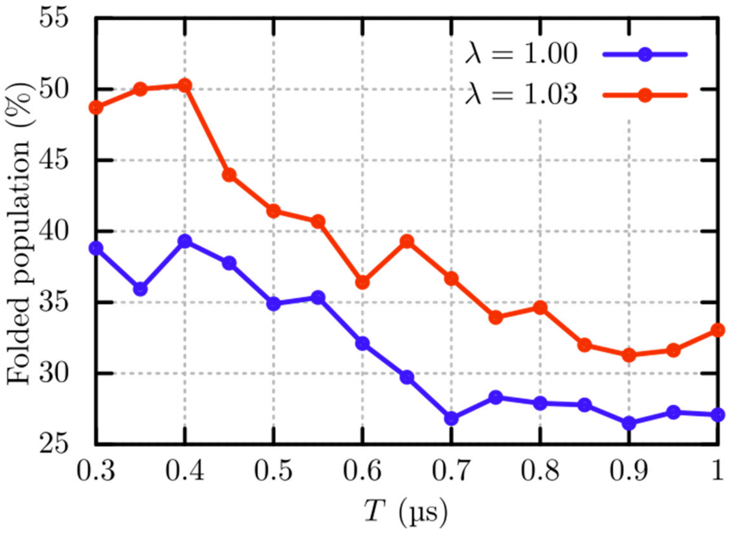

Supplementary Figure S1: Convergence of the folded population for chignolin using different trajectory length, *T*.

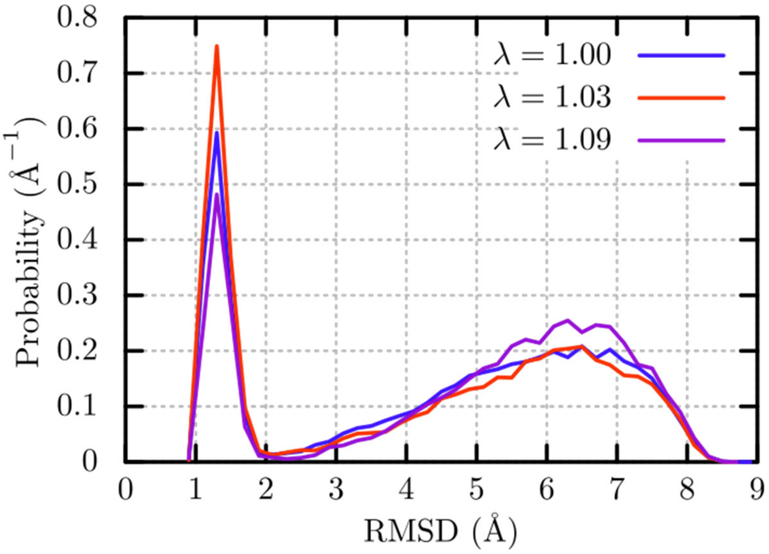

Supplementary Figure S2: Probability densities along the Ca-RMSD of chignolin for different values of l obtained from GaREUS simulations. The profiles for $\lambda = 1.00$ and $\lambda = 1.03$ are computed using the 1-ms simulations, while that for $\lambda = 1.09$ is using the 0.425-ms simulation. The folded population for $\lambda = 1.09$ is 22%.

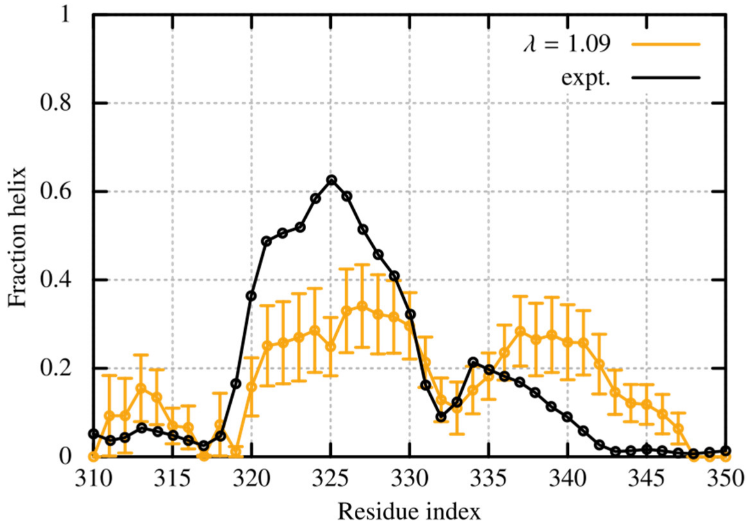

Supplementary Figure S3: Fraction of helix in the C-terminal domain of TDP-43 for l=1.09. The helicity is evaluated through the DSSP algorithm.

(a) $\lambda = 1.00$

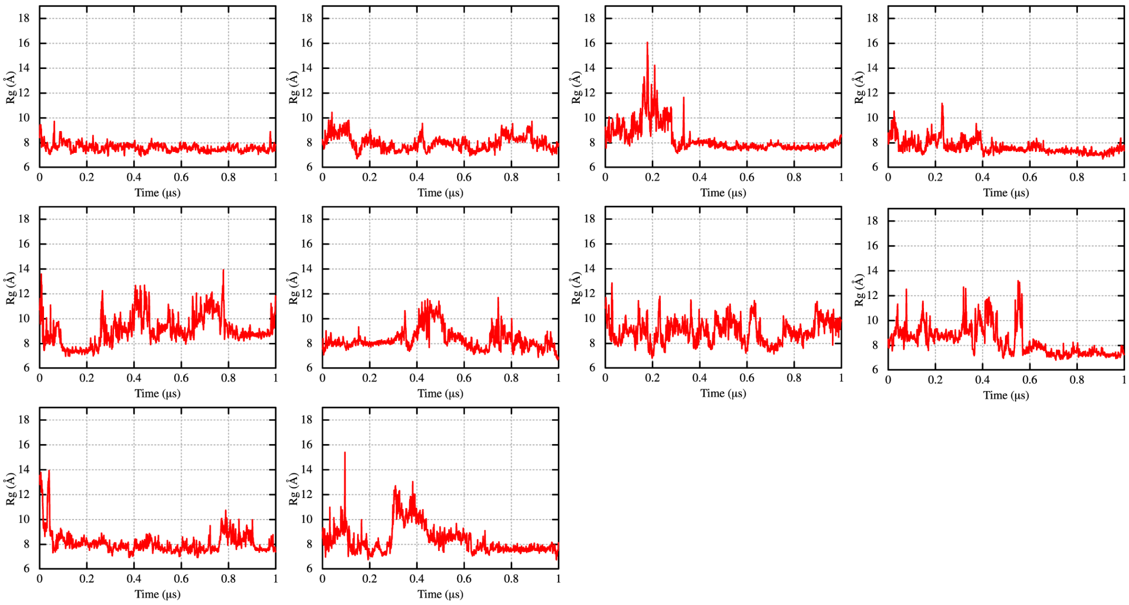

(b) $\lambda = 1.03$

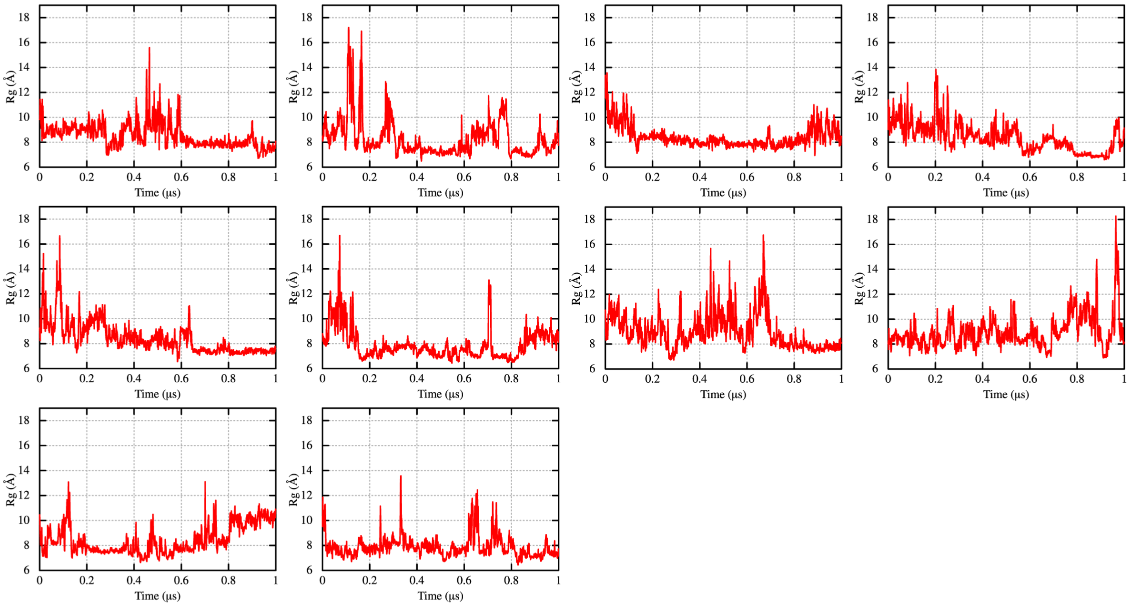

Supplementary Figure S4: The radius of gyration, R_g, of TDP-43. Each plot is obtained from ten independent GaMD simulations for $\lambda = 1.00$ (a) and 1.03 (b).

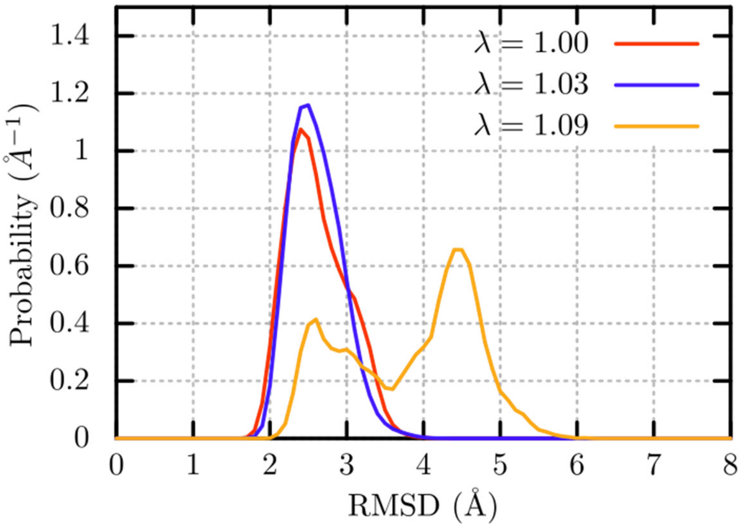

Supplementary Figure S5: Distribution of Ca-RMSD for c-Src kinase. The terminal residues are excluded for the analysis. The profiles are calculated from five independent trajectories for $\lambda = 1.00$ and $\lambda = 1.03$, and from one trajectory for $\lambda = 1.09$.

(a) $\lambda = 1.00$

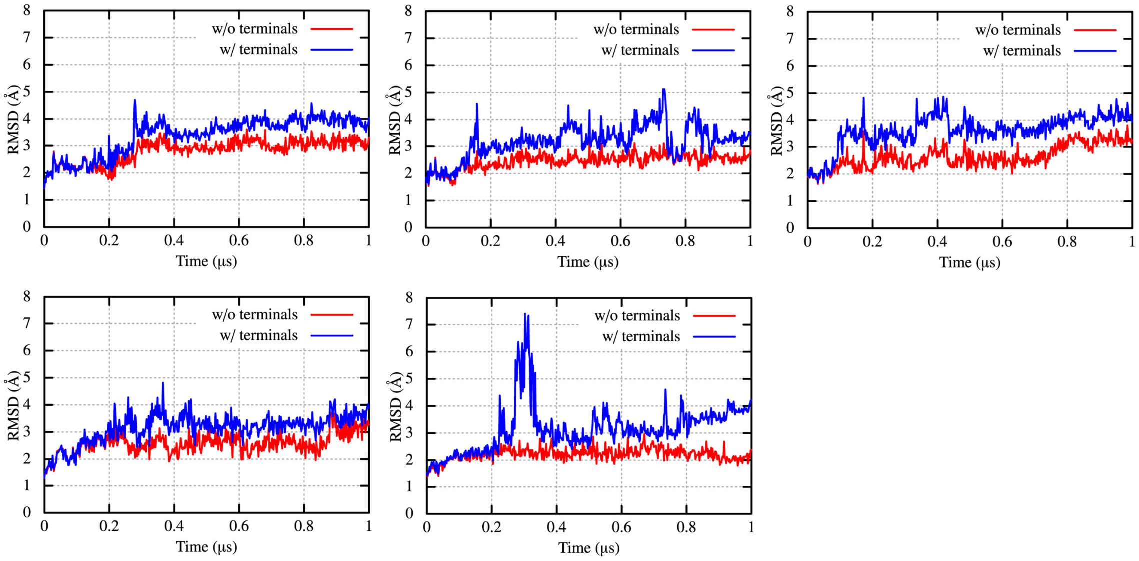

(b) $\lambda = 1.03$

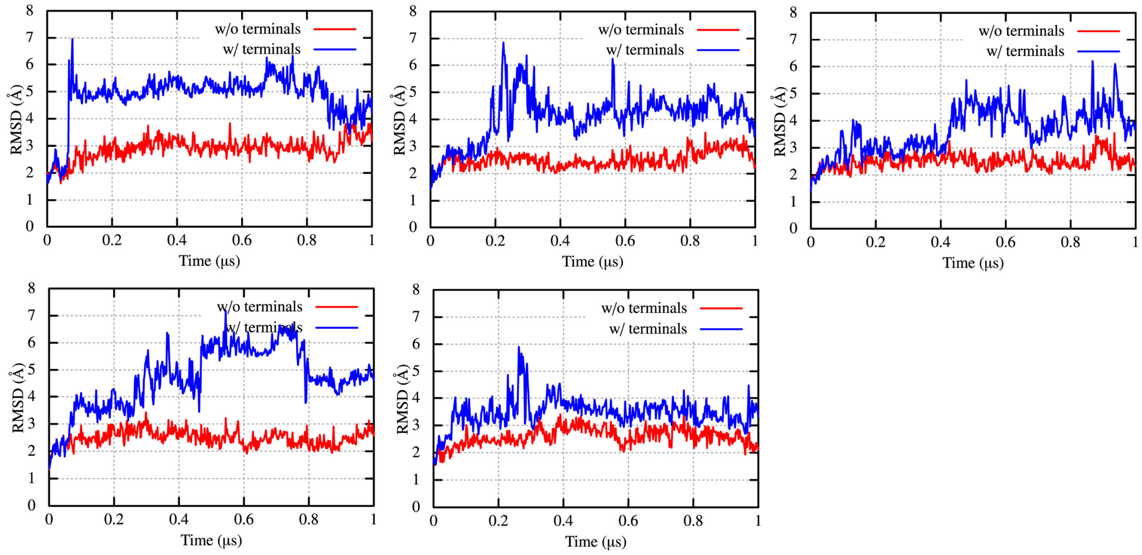

Supplementary Figure S6: The Cα-RMSD of c-Src kinase in dilute solution. Each plot is obtained from five independent simulations for $\lambda = 1.00$ (a) and 1.03 (b). Blue and red lines show the Cα-RMSD with and without 10 terminal residues.

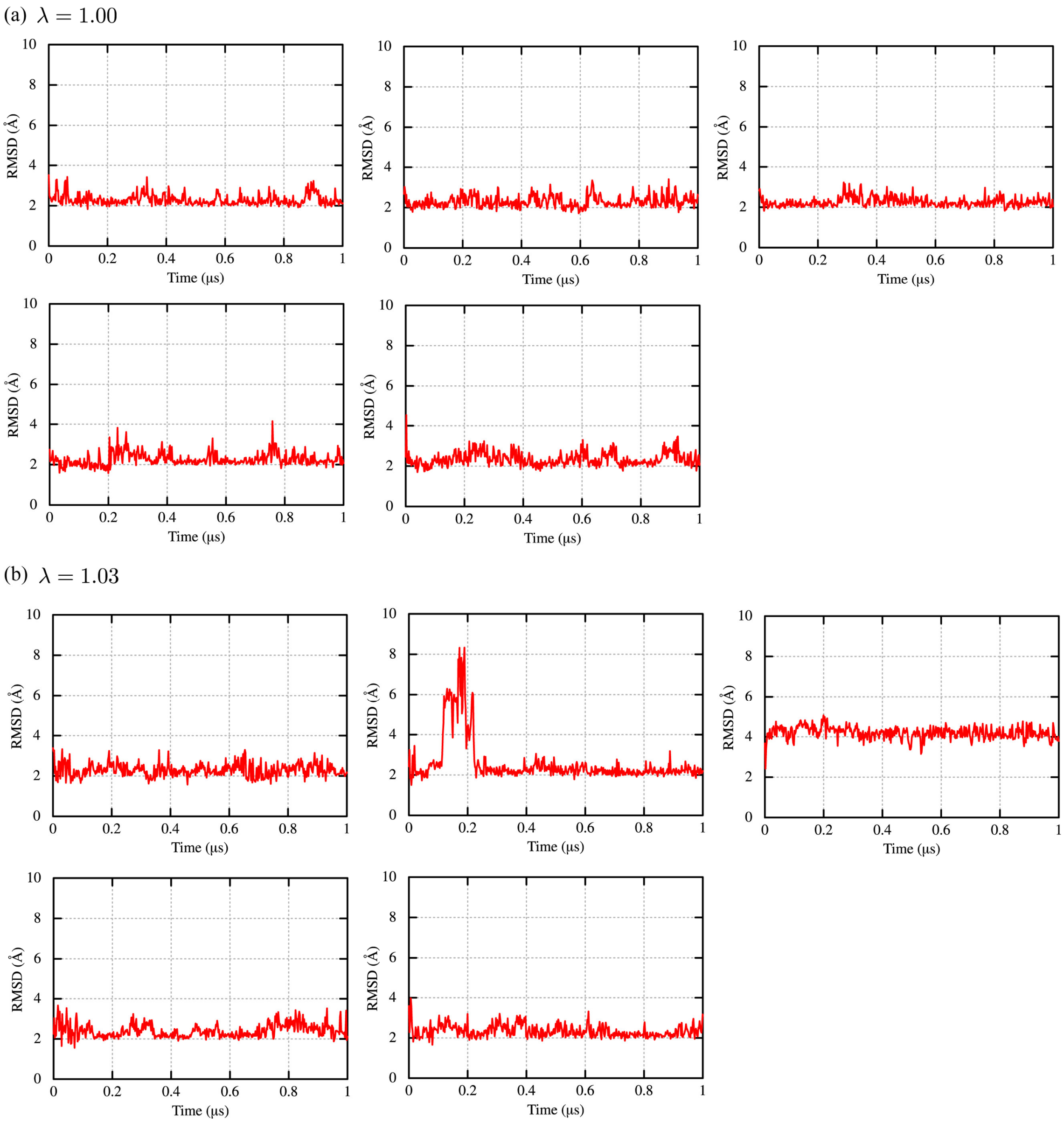

Supplementary Figure S7: The Cα-RMSD of villin in dilute solution. Each plot is obtained from five independent MD simulations for $\lambda = 1.00$ (a) and 1.03 (b).

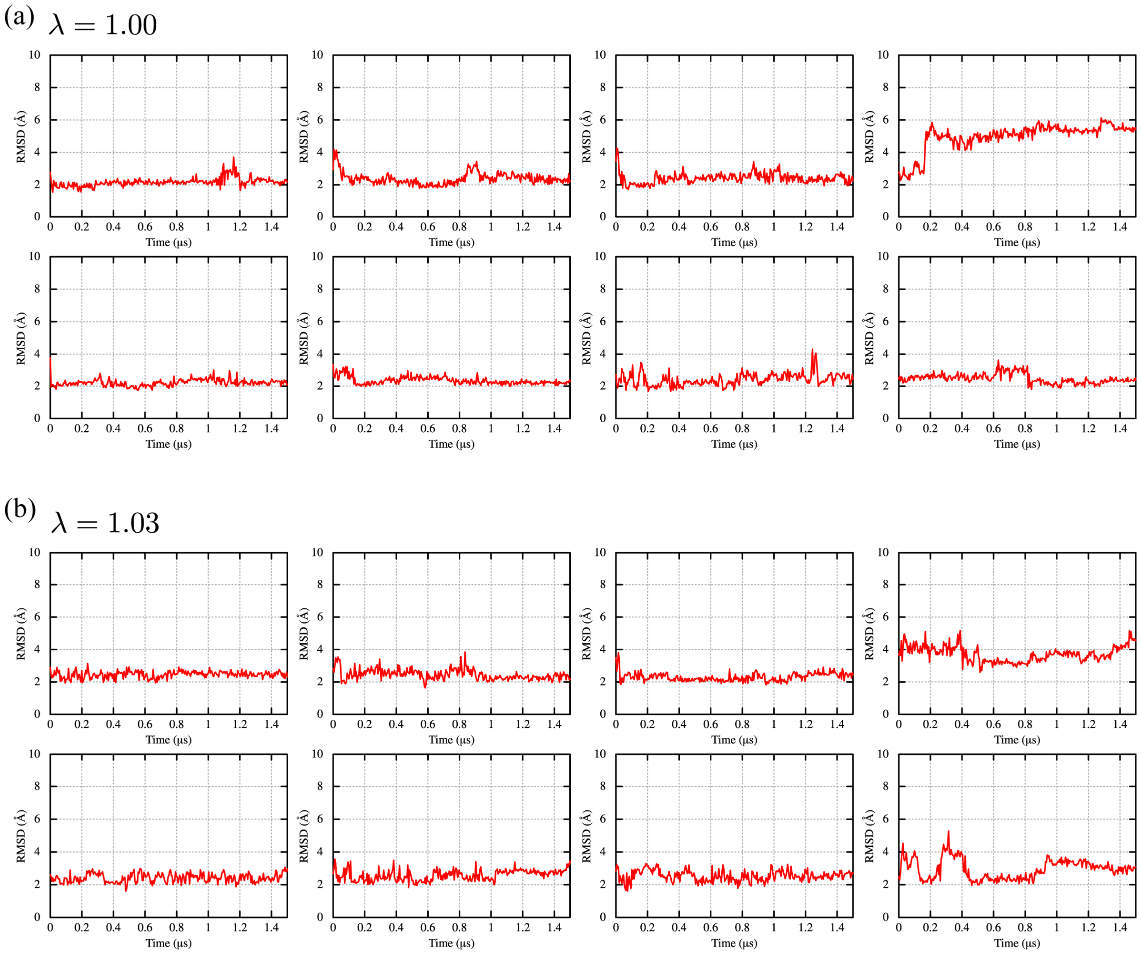

Supplementary Figure S8: The Cα-RMSD of villins in crowded solutions. $\lambda = 1.00$ (a) and 1.03 (b). In each simulation system, eight villins are included and the Cα-RMSD is calculated for the individual protein.

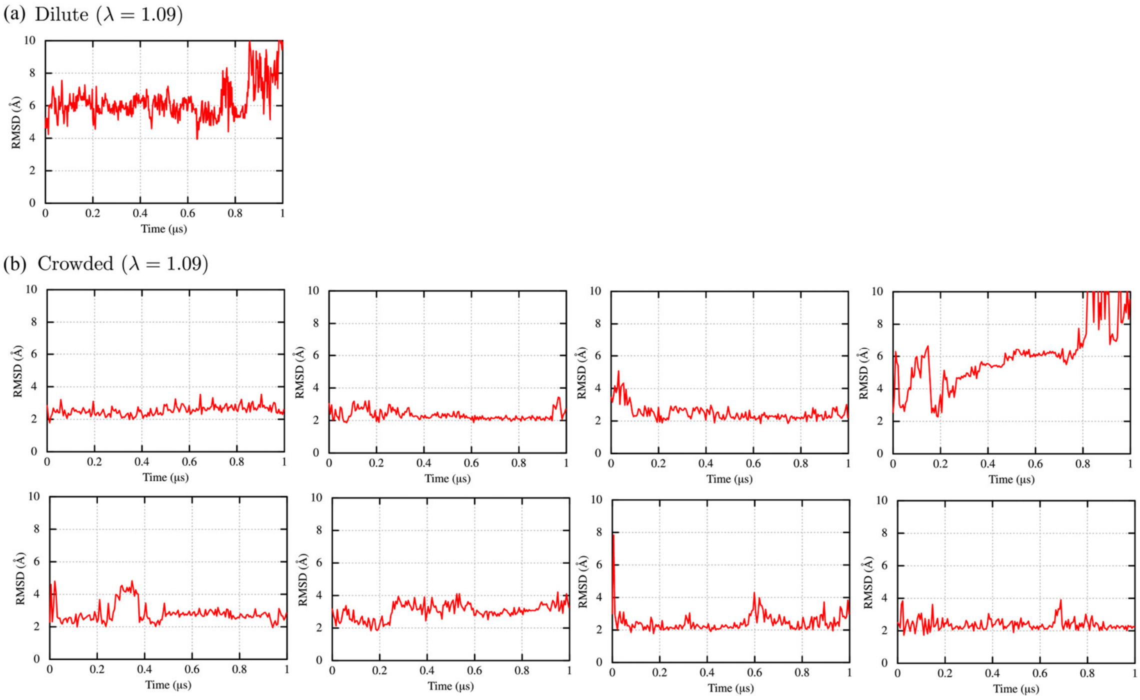

Supplementary Figure S9: The Cα-RMSD of villins for $\lambda = 1.09$ in the dilute (a) and crowded solutions (b). In the crowded solution, eight villins are included and the Cα-RMSD is calculated for the individual protein.

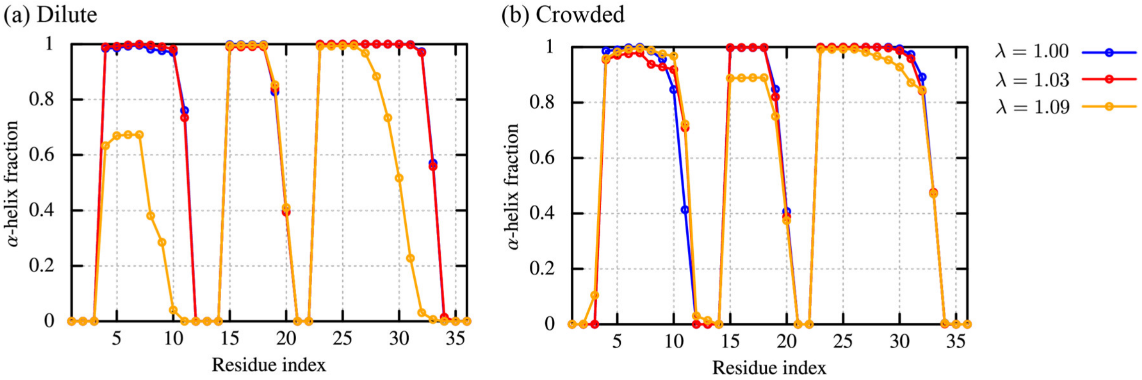

Supplementary Figure S10: The a-helix fraction of villin in the dilute (a) and crowded solutions (b). The fraction is computed using DSSP algorithm. In the case of the dilute solution, the fractions for $\lambda = 1.00$ and 1.03 are averages of the five independent trajectories, while that for $\lambda = 1.09$ is computed from one trajectory. As for the crowded solution, the averages of the fraction over eight villins are shown for all the conditions.
